# A review of the alderfly genus *Leptosialis* Esben-Petersen (Megaloptera, Sialidae) with description of a new species from South Africa

**DOI:** 10.3897/zookeys.201.2623

**Published:** 2012-06-14

**Authors:** Benjamin W. Price, Xingyue Liu, Ferdy C. de Moor, Martin H. Villet

**Affiliations:** 1 Department of Freshwater Invertebrates, Albany Museum, Somerset Street, Grahamstown 6140, South Africa; 2 Department of Zoology & Entomology, Rhodes University, University Road, Grahamstown 6140, South Africa; 3 Current address: Department of Ecology & Evolutionary Biology, University of Connecticut, 75 North Eagleville Road, Storrs, 06269, CT, USA; 4 Department of Entomology, China Agricultural University, Beijing 100193, China

**Keywords:** Sialidae, *Leptosialis*, taxonomy, South Africa

## Abstract

The monotypic South African alderfly genus *Leptosialis* Esben-Petersen, 1920 is reviewed and *Leptosialis africana* Esben-Petersen, 1920 is redescribed. In the process a new species of alderfly *Leptosialis necopinata* **sp. n.** from the Eastern Cape and KwaZulu-Natal provinces of South Africa is recognised and described. Within Sialidae the new species most closely resembles *Leptosialis africana*. A key to the two species of *Leptosialis* using both adult and larval characters is provided.

## Introduction

The Afrotropical Megaloptera fauna have historically received very little attention and only four species of alderfly (Sialidae) are currently recognised: *Sialis vanderweelei* Aspöck & Aspöck, 1983 (Egypt), *Protosialis afra* Navás, 1936 and *Protosialis madegassa* Navás, 1927 (Madagascar), and *Leptosialis africana* Esben-Petersen, 1920 (South Africa). *Leptosialis* Esben-Petersen was established from adult specimens collected in the Cedarberg Mountains of the Western Cape Province. Additional localities and illustrations were provided by Barnard (1931, 1940). Crass’s (1949) description of putative larvae of *Leptosialis africana* used specimens collected in the Eastern Cape and KwaZulu-Natal provinces, approximately 1000 km from the type locality.

With very few specimens in museum collections, it is clear that these African alderflies are rarely encountered insects. The adults have historically been collected in summer amongst riparian vegetation associated with the still reaches of slow-flowing streams, and the larvae inhabit slow flowing streams with clay or silt substrates (Crass 1949, Mansell 2003).

While examining the Iziko South African Museum and Albany Museum collections, two distinct phenotypes of putative *Leptosialis africana* adults were found, corresponding to the two disjunct regions from which they have been recorded. The specimens from the Eastern Cape and KwaZulu-Natal provinces are distinct enough from Western Cape *Leptosialis africana* to warrant recognition as a new species. Crass’s (1949) larval material from the Eastern Cape and KwaZulu-Natal correspond to the new species.

## Materials and methods

The specimens included in the present study are deposited in three collections: the Albany Museum, Grahamstown (AMGS), the Natural History Museum, London (NHM), and the Iziko South African Museum, Cape Town (SAMC). Label data for the specimens are presented in quotation marks; information from different labels is separated by a virgule (/), and information on different lines of a label is separated by commas. Information in square brackets clarifies or augments the often cryptic text on the specimen label(s) and provides their geographic coordinates.

Terminalia preparations were made by clearing the distal half of the abdomen in a cold, saturated potassium hydroxide (KOH) solution for 8–10 h. After neutralising the KOH with acetic acid and water, the distal half of the abdomen was transferred to glycerine for further dissection and examination. Following examination, the cleared terminalia was placed in a microvial containing glycerine and pinned beneath the specimen. The terminologies of venation and terminalia follow Wootton (1979) and Aspöck and Aspöck (2008), respectively. Wing and body measurements were made from photographs of whole specimens with reference to the photographed scale bar.

## Taxonomy

### Genus Leptosialis

Esben-Petersen

http://species-id.net/wiki/Leptosialis

Leptosialis Esben-Petersen, 1920: 502. Type species: *Leptosialis africana* Esben-Petersen, 1920: 502 (original designation).

#### Diagnosis.

The adults of *Leptosialis* are characterized by the following morphological traits: the narrowly elongated forewing, which is about 3.0–4.0 times longer than wide; the distally branched Rs; the MA either unbranched, bifurcated or trifurcated; the MP distally branched or with one or both main branches bifurcated; the male 9^th^ tergum with a pair of posterolateral, digitiform processes; the paired ectoproct; the 11^th^ gonocoxite in caudal view ventrally with a pair of acute, hook-like processes. At present no characters can be used to distinguish the larvae of the genus *Leptosialis* from other alderfly genera, many of which currently lack larval description.

#### Description.

##### Adults (Figs 1–4).

Forewing length ~8–11 mm in males; ~11–12 mm in females. Body: generally brown or blackish-brown. Head: antenna pilose approximately half the length of the forewing; ocelli absent, or represented by three very small tubercles on vertex; labrum ~4.0–5.0 times wider than long, lateral margins rounded, front margin slightly emarginated. Prothorax > two times wider than long. Legs: yellow or dark brown, bearing dense setae; tarsal claws reddish brown. Wings: Forewing 3.0-4.0 times longer than wide, minutely hirsute, margins pilose; costal area broadened basally; subcostal area with five to eight distinct costal crossveins proximally; sc-r absent; Rs distally branched, MA either unbranched, bifurcated or trifurcated, MP distally branched or with one or both main branches bifurcated, CuA bifurcated; three or four crossveins between R and Rs. Hindwing as broad or slightly broader than forewing, about 3.0 to 3.5 times as long as wide; two or three distinct costal crossveins proximally; venation similar to forewing, with three crossveins between R and Rs. Male genitalia: 9^th^ tergum transversely arched, with a pair of posterolateral, digitiform processes; ectoproct paired, small, roundly inflated ventrad; 11^th^ gonocoxite in caudal view ventrally with a pair of acute, hook-like processes. Female genitalia: 7^th^ sternum broad, posterior margin distinctly produced; 9^th^ gonocoxite broad, apex bearing small, stout gonostylus.

##### Larvae (Figs 22–23).

Head: yellow to reddish brown. Thorax: pro-, meso- and metathorax orange to reddish brown, with distinct reticulated patterns of yellowish marks. Legs: pale yellow, bearing dense setae; tarsal claws reddish brown; Abdomen: dark purplish or blackish brown dorsally with paired, pale, submedian, comma-shaped marks on each segment; anal prolegs and hooked claws absent; elongated caudal filamentous appendage present; 7 pairs of pale yellow lateral abdominal gills present.

#### Remarks.

The genus *Leptosialis* is the only representative of the Sialidae in South Africa. Because wing venation is quite different between the two *Leptosialis* species, it is difficult to find a stable morphological diagnosis for this genus. The male ninth tergum with a pair of digitiform processes could be the most important character to distinguish *Leptosialis* from its closely related genera, *Stenosialis* and *Austrosialis*.The two species may be distinguished using the following key.

**Figures 1–4.**
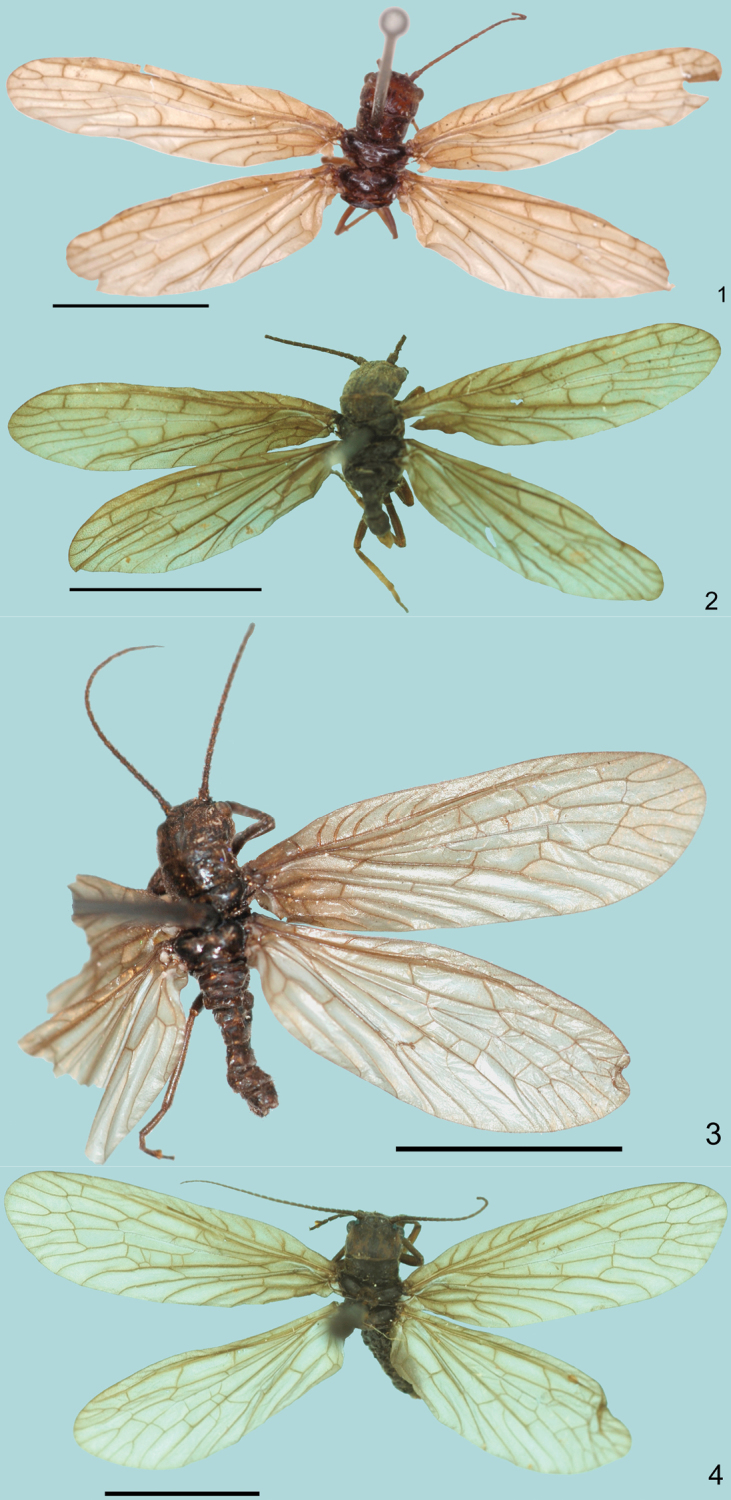
Habitus images of *Leptosialis* species. **1** *Leptosialis africana* Esben-Petersen, female, holotype **2** same, male **3** *Leptosialis necopinata* sp. n. male, holotype **4** same, female. Scale bars = 5.0 mm

#### Key to *Leptosialis*

**Table:** 

1	Adults: (Figs 1, 2) Forewing narrowly rounded with MA and both branches of MP simple (Barnard 1931: fig. 12). Male: terminalia with 11^th^ gonocoxite broadly triangular dorsoventrally, with a pair of short, simple hook-like processes ventrally, visible in caudal view. Female: (Fig. 15) 8^th^ gonocoxite small, subtriangular. Larvae: (Fig. 22) Head reddish brown; pro-, meso- and metathorax reddish brown, distinctly with reticulated patterns of yellowish marks; abdomen blackish brown	*Leptosialis africana*
–	Adults: (Figs 3, 4) Forewing broadly rounded with MA branches bi- and trifurcated and with one or both branches of MP bifurcated (Fig. 9). Male: terminalia with 11^th^ gonocoxite narrowly triangular dorsoventrally, with a pair of long, hook-like processes with additional subterminal hook ventrally, visible in caudal view (Figs 18-19). Female: 8^th^ gonocoxite large, terminally divided to form a pair of subtriangular lobes, obtusely protruding posterolaterally (Fig. 21). Larvae: (Fig. 23) Head orange, slightly darkened medially on frons; prothorax orange, dorsally with indistinct marks, meso- and metathorax castaneous with reticulated yellowish marks; abdomen dark purplish-brown with paired, pale, submedian, comma-shaped marks on each segment (Fig. 1: Crass 1949)	*Leptosialis necopinata* sp. n.

### Leptosialis africana

Esben-Petersen

http://species-id.net/wiki/Leptosialis_africana

Figures 1–2 5–6 10–15 22

Leptosialis africana Esben-Petersen, 1920: 502. Type locality: South Africa (Great Winterhoek Mountains).

#### Type material.

Holotype, male [see remarks below] (pinned; Fig. 1), South Africa: Western Cape: “G[rea]t. Winterhoek Mts., Tulbagh [33°8'S, 19°10'E], XI.1916, K.H. Barnard / SAM-MEG-A000041 / Holotype” (SAMC).

#### Other material.

1 male (pinned, Fig. 2), South Africa: Western Cape: “G[rea]t. Winterhoek Mts., Tulbagh, [33°8'S, 19°10'E], XI.1932, K.H. Barnard / SAM-MEG-A000042” (SAMC); 1 female (pinned), South Africa: Western Cape: “Hottentots Holland Mts., Steenbras [34°6'S, 18°58'E], XI.1932, K.H. Barnard, SAM-MEG-A000043” (SAMC); 1 male (pinned), South Africa: Western Cape: “Caledon Distr., R. Zonder End, Oudebosch [34°9'S, 19°54'E], XII.1919, K.H. Barnard / 1930-131” (NHM); 2 larvae (in alcohol), South Africa: Western Cape: “Bettys Bay, Small Vlei [34°21'S, 18°56'E], 18.I.1957, [A.D. Harrison and J.D. Agnew] / FRW 128C” (AMGS).

#### Diagnosis.

The adults of *Leptosialis africana* may be easily distinguished from adults of *Leptosialis necopinata* sp. n. by the shape and venation of the forewing, where the wings of *Leptosialis africana* are narrowly rounded compared to being broadly rounded to truncated in *Leptosialis necopinata* sp. n. In addition, the wings of *Leptosialis africana* show MA and both branches of MP all simple,while *Leptosialis necopinata* sp. n. has MA and one or both branches of MP all bearing additional forks. The larvae of *Leptosialis africana* may be distinguished by the reddish brown head and thorax dorsally with distinct yellowish marks that are lacking in *Leptosialis necopinata* sp. n.

#### Description.

##### Adult male (Fig. 2).

**Figures 5–8.**
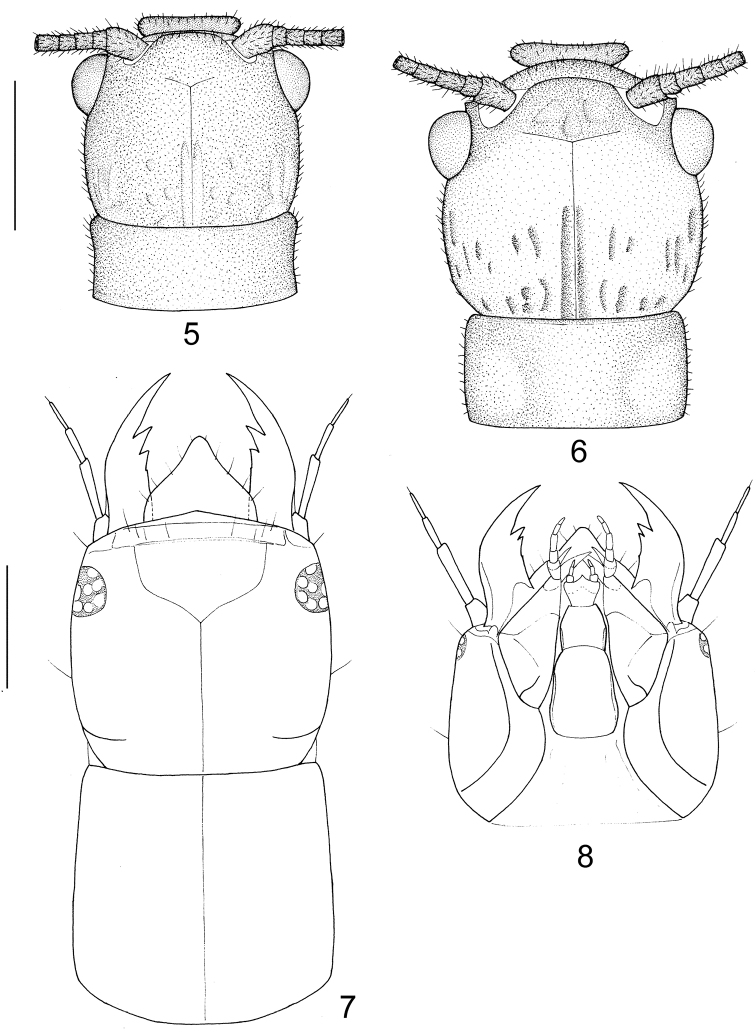
Head and prothorax of *Leptosialis*. **5** *Leptosialis africana*, head and pronotum of male adult, dorsal view **6** same of female adult, dorsal view **7** *Leptosialis necopinata* sp. n., head and pronotum of larva, dorsal view **8** same, head of larva, ventral view. Scale bar = 1.0 mm.

Forewing length 8.1 mm, hindwing length 7.0 mm (n = 1). Head (Fig. 5): pale to dark brown, frons and clypeus black; vertex with a pair of raised black vittae medially and several small, raised, black protrusions laterally. Compound eyes blackish brown, strongly produced. Antennae dark brown, pilose, approximately half the length of the forewing. Mouthparts yellowish brown, diminutive. Thorax (Fig. 5): entirely blackish brown. Legs yellow, bearing dense yellowish setae; coxae blackish brown; femora pale brown; 1^st^-3^rd^ tarsomeres distally pale brown; 4^th^ and 5^th^ tarsomeres brown; tarsal claws reddish brown. Wings narrowly rounded, brown, slightly darker proximally; veins brown. Forewing nearly 4.0 times as long as wide; five to eight distinct costal crossveins proximally; sc-r absent; Rs distally branched, MA unbranched, MP distally branched, and CuA bifurcated; three or four crossveins between R and Rs. Hindwing slightly broader than forewing, about 3.5 times as long as wide; two or three distinct costal crossveins proximally; venation similar to forewing, with three crossveins between R and Rs. Abdomen: blackish brown. Terminalia (Figs 10–13) with 9^th^ tergum transversely arched, with a pair of digitiform processes posterolaterally that curve slightly ventromedially; anterior and posterior margins slightly concave arcuately in dorsal view; 9^th^ sternum slightly longer than 9^th^ tergum; posterior margin moderately produced; 9^th^ gonocoxite in lateral view nearly elliptical; ectoproct paired, small, roundly inflated ventrad; 11^th^ gonocoxite broadly triangular dorsoventrally, dorsal margin slightly sinuous in dorsal view, with a pair of short and acutely hook-like processes ventrally in caudal view.

##### Adult female (Figs 1, 6).

Forewing length 11.1 mm, hindwing length 10.2 mm (n = 2). Larger than male, but similar in colouration and venation. Terminalia (Figs 14-15) with 7^th^ sternum broad, posterior margin distinctly produced; 8^th^ gonocoxite rather small, subtriangular; 9^th^ gonocoxite broad; apex with small, stout gonostylus, ectoproct feebly sclerotized, small, suboval.

##### Larva (Fig. 22).

Head reddish brown; thorax reddish brown, distinctly marked with complicate patterns of yellowish marks; legs pale yellow, bearing dense setae, tarsal claws reddish brown. Abdomen blackish brown dorsally with pale yellow lateral abdominal gills.

#### Distribution.

South Africa: Western Cape (Fig. 24).

#### Remarks.

The type specimen was noted by Esben-Petersen in his description as male, but its abdomen and genitalia are now lost. This species of alderfly shows sexual dimorphism, with females being larger than males. Although it lacks an abdomen, the holotype is probably a female, based on comparison with confirmed male and female specimens, and contrary to the original description.

**Figure 9.**
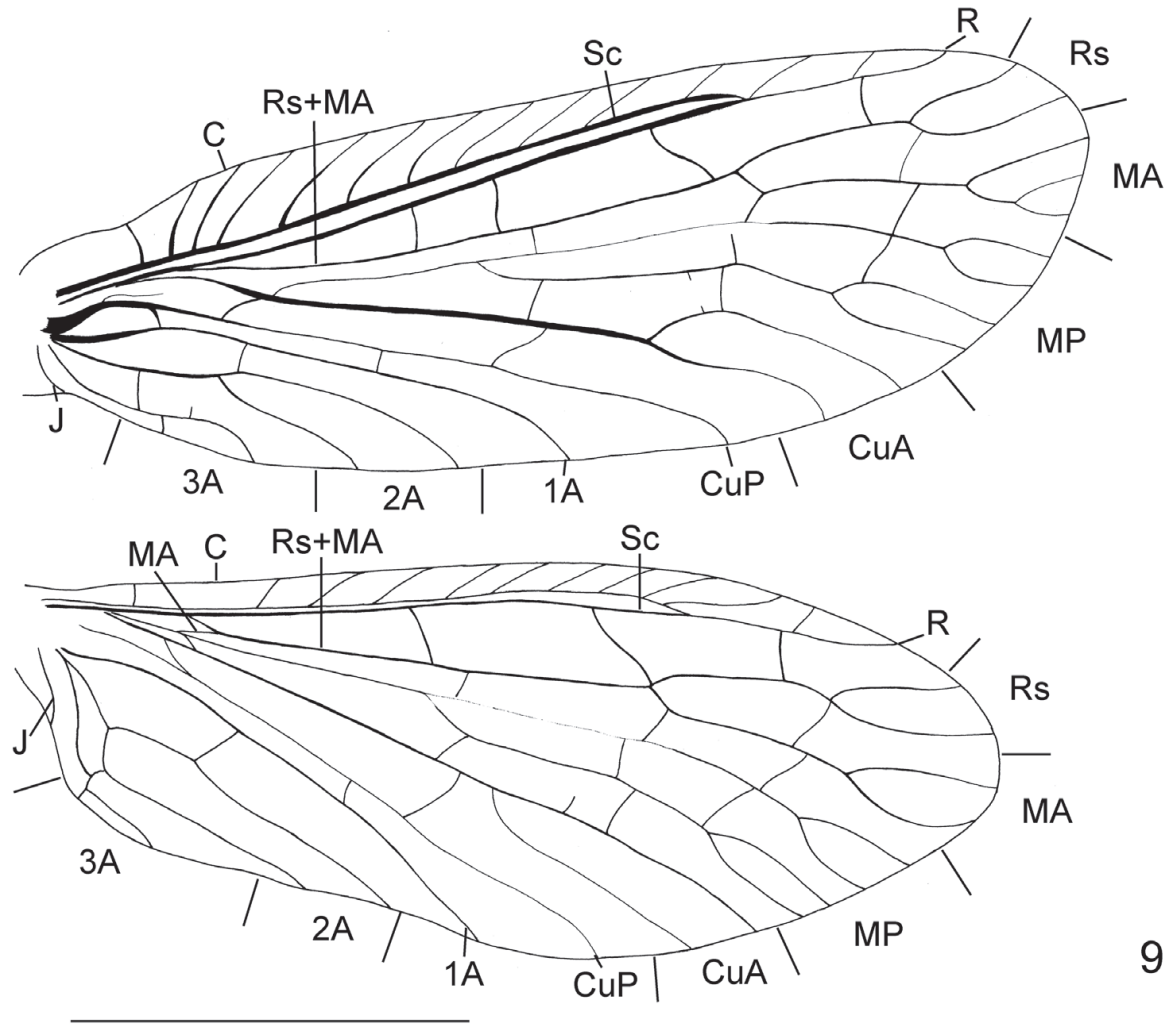
Wing venation of *Leptosialis necopinata* sp. n. Scale bar = 5.0 mm.

**Figures 10–15.**
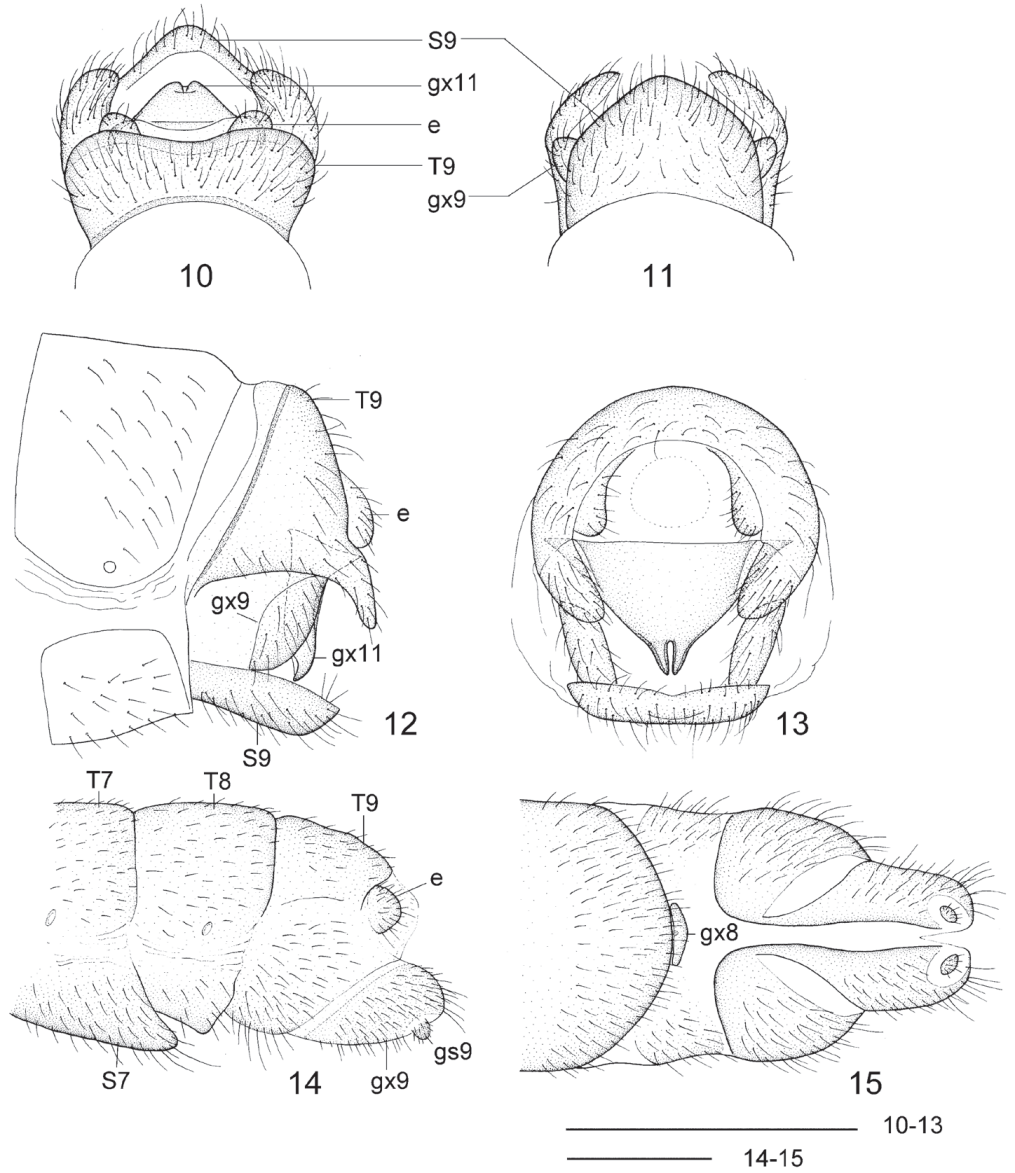
*Leptosialis africana* Esben-Petersen. **10** male terminalia, dorsal view 11 male terminalia, ventral view **12** male terminalia, lateral view **13** male terminalia, caudal view **14** female terminalia, lateral view **15** female terminalia, ventral view. T7-9: seventh to ninth tergum; S7-9: seventh to ninth sternum; gx8, 9 **11** eighth, ninth and eleventh gonocoxite; gs9: ninth gonostylus; e: ectoproct. Scale bars = 0.5 mm.

### Leptosialis necopinata

Price, Liu, de Moor & Villet sp. n.

urn:lsid:zoobank.org:act:0DC77737-04AC-49B5-A2CF-3B2A09201DEA

http://species-id.net/wiki/Leptosialis_necopinata

Figures 3–4 7 -9 16–21 23

#### Type locality.

South Africa: KwaZulu-Natal: Kokstad [30°33'S, 29°25'E].

#### Type material.

Holotype, male (pinned; Fig. 3), SOUTH AFRICA: KwaZulu-Natal: “Kokstad [30°33'S, 29°25'E], I.1941, [possibly R.S. Crass] / GEN 2081A / HOLOTYPE” (AMGS). Paratypes, 1 male, 5 female (all pinned), same data as holotype, male “GEN 2081B / PARATYPE” females “GEN 2081C / PARATYPE” (AMGS).

#### Other material.

SOUTH AFRICA: 1 female (pinned), Eastern Cape: “Maclear Municipal Dam [31°4'0"S, 28°18'36"E], 27.III.1993 [F.C. de Moor and H.M. Barber-James] / ECR 124S” (AMGS); 2 female (pinned), KwaZulu-Natal: “Tugela river [30°6'0"S, 29°11'24"E], 15.XI.1959, [M. Chutter] / GEN 380H” (AMGS); 3 larvae (in alcohol), KwaZulu-Natal: “Mooi River [29°15'0"S, 29°58'12"E], 15.VI.1995, [C.W.S. Dickens] / MOI 52K” (AMGS); 4 larvae (in alcohol), KwaZulu-Natal: “Mooi River [29°21'36"S, 29°53'24"E], 4.I.1996, [F.C. de Moor, C.W.S. Dickens & R.S. Crass] / MOI 69H” (AMGS).

#### Etymology.

The specific epithet ‘*necopinata*’ is a feminine adjective in the second declension which refers to the Latin for unexpected or unforeseen, following the discovery of the adults strikingly different from *Leptosialis africana* and relating to the unforeseen occurrence of this second species in the relatively well-sampled waters of South Africa.

#### Diagnosis.

The adults of *Leptosialis necopinata* sp. n. may be easily distinguished from adults of *Leptosialis africana* in having broadly rounded to truncated wings compared to narrowly rounded wings in the latter species. In addition the forewing venation of *Leptosialis necopinata* sp. n. has MA and one or both branches of MP all forked distally, whereas those of *Leptosialis africana* are fused into three simple veins. The larvae of *Leptosialis necopinata* sp. n. can be distinguished from *Leptosialis africana* by the paler colouration of head and thorax, which lack distinct marks on the vertex and pronotum.

#### Description.

##### Adult male (Fig. 3).

Forewing length 10.6 mm, hindwing length 9.8 mm (n = 2).

**Figures 16–21.**
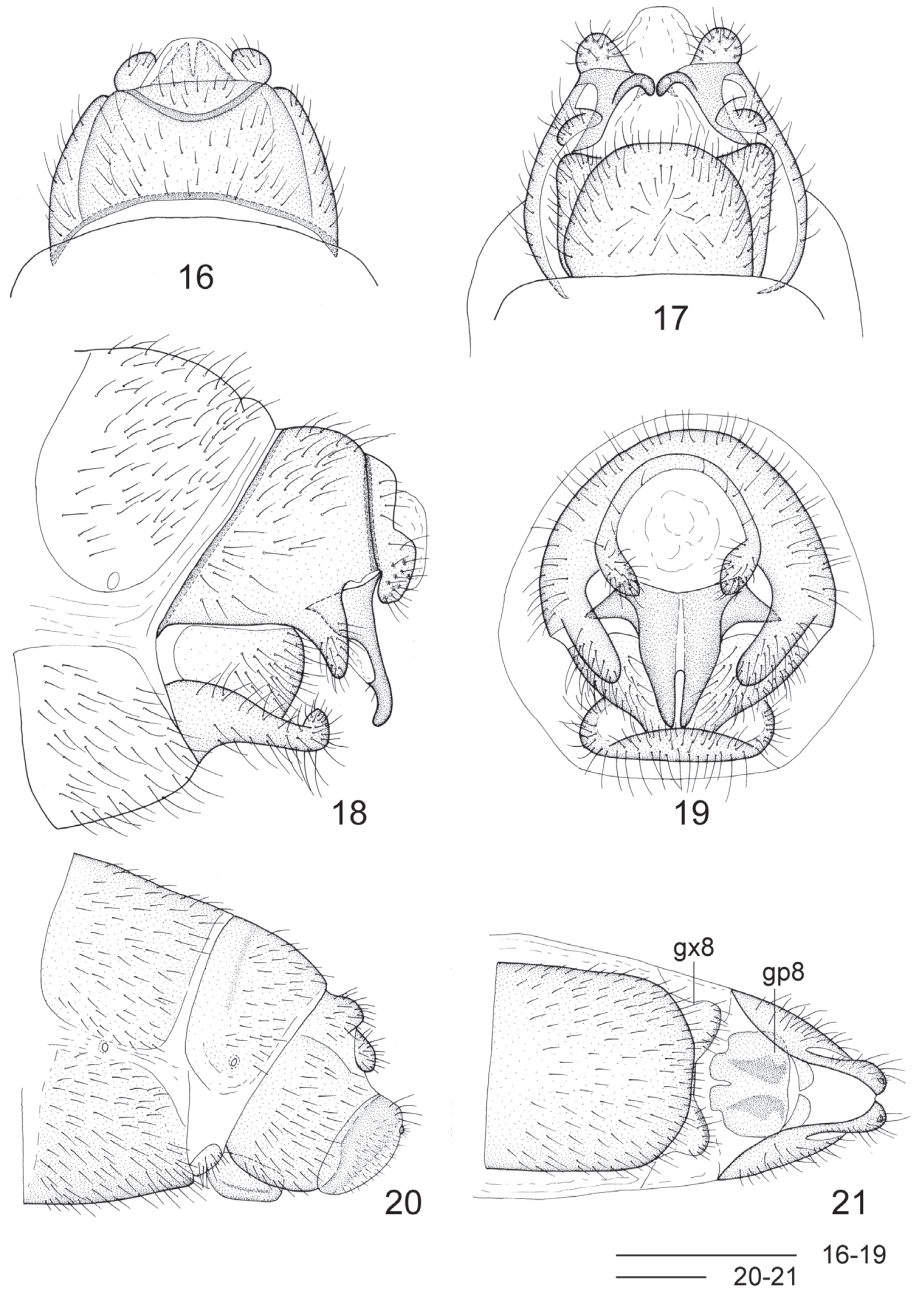
*Leptosialis necopinata* sp. n. **16** male terminalia, dorsal view **17** male terminalia, ventral view **18** male terminalia, lateral view; **19** male terminalia, caudal view **20** female terminalia, lateral view **21** female terminalia, ventral view. gx8: eighth gonocoxite; gp8: eighth gonapophyses. Scale bars = 0.5 mm.

Head (Fig. 7) black, slightly pale brown surrounding posterior margin of compound eyes; vertex with a pair of raised black vittae medially, and several small raised black protrusions laterally Compound eyes blackish brown, strongly produced. Antennae blackish brown, pilose, approximately half the length of the forewing. Mouthparts blackish brown. Thorax (Fig. 8) entirely black. Legs dark brown throughout, bearing dense brown setae; tarsal claws reddish brown. Wings (Fig. 9) distally broadly rounded to truncate, brown, slightly darker proximally; veins brown. Forewing approximately 3.0 times as long as wide; proximally with five to eight distinct costal crossveins; sc-r absent; RS distally branched, MA bifurcated or trifurcated, MP with one or both main branches bifurcated, and CuA bifurcated, three or four crossveins between R and Rs. Hindwing as broad as forewing, about 3.0 times as long as wide; with two or three distinct costal crossveins proximally; venation similar to forewing, with three crossveins between R and Rs. Abdomen blackish brown. Terminalia (Figs 16–19) with 9^th^ tergum transversely arched, with a pair of digitiform processes posterolaterally that curve slightly ventromedially; anterior and posterior margins slightly arcuately concave in dorsal view; 9^th^ sternum slightly longer than 9^th^ tergum, posterior margin moderately produced; 9^th^ gonocoxite in lateral view nearly elliptical; ectoproct paired, small, roundly inflated ventrad; 11^th^ gonocoxite broadly triangular dorsoventrally, dorsal margin slightly sinuous in dorsal view, ventrally with a pair of acute hook-like processes in caudal view, in lateral view ventral processes with an anteriorly directed, hook-shaped accessory protuberance one third along its length.

##### Adult female (Fig. 4).

Forewing length 11.7 mm, range 11–12 mm, hindwing length 10.4 mm, range 10–11 mm (n = 5). Larger than male, but similar in colouration and wing venation. Head pale to dark brown, with frons and clypeus black; vertex with a pair of raised black vittae medially, and several small raised black markings laterally. Terminalia (Figs 20–21) with 7^th^ sternum broad, with posterior margin feebly produced; 8^th^ gonocoxite almost separated into a pair of subtriangular lobes, which are obtusely protruding posterolaterally; 9^th^ gonocoxite broad, apex with small, stout gonostylus; ectoproct feebly sclerotized, small, suboval.

##### Larva

Head (Figs 7, 8, 23) yellow, slightly darkended medially on frons. Prothorax (Fig. 7) yellow to orange, dorsally with indistinct markings, meso- and metathorax castaneous with reticulated yellowish markings; Legs pale yellow, bearing dense setae, tarsal claws reddish brown; Abdomen dark purplish-brown dorsally with paired, pale, submedian, comma-shaped marks on each segment; lateral abdominal gills pale yellow.

#### Distribution.

South Africa: Eastern Cape and KwaZulu-Natal provinces (Fig. 24).

#### Remarks.

The larvae of *Leptosialis necopinata* sp. n. have been described in detail by Crass (1949).

**Figures 22–23.**
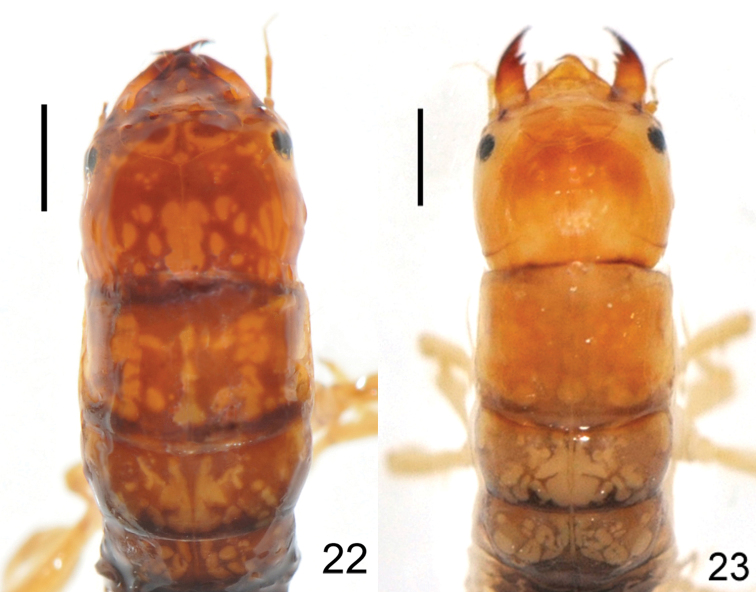
*Leptosialis* larvae, showing color patterns on head and thorax **22** *Leptosialis africana* **23** *Leptosialis necopinata* sp. n. Scale bar = 1.0 mm.

**Figure 24.**
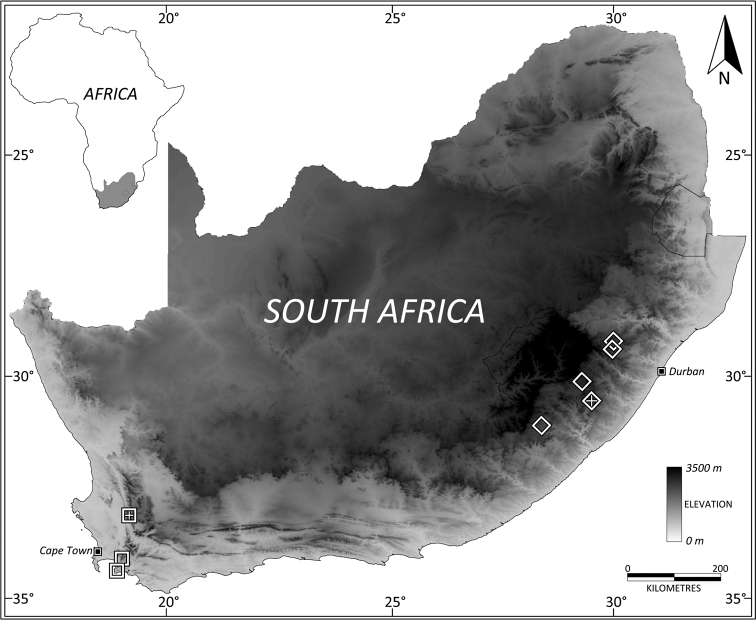
Distribution map of specimens housed in the Iziko and Albany Museums in South Africa. *Leptosialis africana*: open squares, *Leptosialis necopinata*sp. n.: open diamonds. Type localities for each species indicated with “+”. Shading indicates elevation.

## Discussion

At first glance *Leptosialis necopinata* sp. n. appears to belong to the Australian endemic genus *Stenosialis* (Liu et al. 2008). However, the male genitalia of *Leptosialis necopinata* sp. n. are generally similar in structure to those of *Leptosialis africana*, confirming the taxonomic status of the new species within the genus *Leptosialis*. Although the forewing venation of *Leptosialis necopinata* sp. n. is similar to that of *Stenosialis* and *Austrosialis* based on the two-branched Rs, 2 or 3-branched MA, and 4-branched MP, the hindwing of *Leptosialis necopinata* sp. n. also has a four-branched MP, which is different from the always three-branched MP in both Australian genera (Liu *et al*. 2008). The diagnosis of the venation in *Leptosialis necopinata* sp. n. does not provide synapomorphies for *Leptosialis* because it may be a plesiomorphic ground plan of sialid venation, which is very similar to the earliest alderfly fossil, *Dobbertinia reticulata* Handlirsch, from the Lower Jurassic of Germany (Ansorge 2001). The venation of Sialidae genera is very conserved and indicative of symplesiomorphies (Flint 1973). A world revision of the genera using genitalic differences is needed to confirm placement of the genera and species. The possible synapomorphic character of *Leptosialis* might be the male 9^th^ tergum with a pair of digitiform posterior projections, although this character is also present in an extinct alderfly species *Protosialis herrlingi* Wichard (2002) from the Eocene Baltic amber. Further clarification on the phylogenetic status of *Leptosialis* awaits a rigorous study of the phylogeny of the world Sialidae.

## Supplementary Material

XML Treatment for Leptosialis

XML Treatment for Leptosialis africana

XML Treatment for Leptosialis necopinata

## Appendix

Localities of specimens housed in the Iziko and Albany Museums in South Africa. (doi: 10.3897/zookeys.201.2623.app). File format: Comma Separates List (csv).
